# Development and validation of the nurses sexual harassment scale in Iran

**DOI:** 10.1186/s12912-024-01759-6

**Published:** 2024-02-08

**Authors:** Maryam Zeighami, Alireza Malakoutikhah, Parvin Mangolian Shahrbabaki, Kamlah Al-Oliamat, Mahlagha Dehghan

**Affiliations:** 1 Department of Nursing and Midwifery, Islamic Azad University, Kerman Branch, Kerman, Iran; 2 Nursing Research Center, Kerman University of Medical Sciences, Kerman, Iran; 3 Obstetric and Gynecology Nursing, Zarqa University, Zarqa, Jordan; 4 Reproductive Health, Family and Population Research Center, Kerman University of Medical Sciences, Haft-Bagh Highway, Kerman, Iran

**Keywords:** Sexual harassment, Scale, Psychometrics, Nurses, Analysis

## Abstract

**Introduction:**

In recent years, the prevalence of sexual harassment has become a global problem, and nursing, like other professions, has not been immune to this issue. By having a valid and reliable instrument, healthcare personnel can be helped in preventing and managing this problem and reduce its negative consequences on mental health and well-being. The aim of this study was developing and psychometrically measuring the Nurses Sexual Harassment Scale.

**Materials and methods:**

This study is the second phase of a mixed method study. Initially in the first phase, a qualitative approach with conventional content analysis was used to explain nurses’ experiences of sexual harassment in the workplace. To design the Nurses Sexual Harassment Scale, qualitative data and literature were reviewed. In the quantitative phase (second phase), the target scale was psychometrically evaluated using content validity (14 experts), face validity (12 nurses with being sexually harassed), construct validity (316 nurses working in hospitals affiliated to Kerman University of Medical Sciences), and reliability (internal consistency and repeatability).

**Results:**

A 15-item scale with two components: “latent sexual harassment” (9 items) and “manifest sexual harassment” (6 items), which explained 68.4% of the total variance was developed. Also, due to the strong correlation between the Nurses Sexual Harassment Scale and the Impact of Event Scale-Revised (*r* = 0.67), convergent validity was confirmed. Also, the scale of the present study had good reliability (Cronbach’s alpha coefficient = 0.94, Omega coefficient = 0.94, and ICC = 0.92).

**Conclusion:**

Given the importance of sexual harassment among nurses, measuring the dimensions of this problem may allow professionals to plan interventions to prevent it. Overall, the results of the present study showed that the psychometric properties of the " Nurses Sexual Harassment Scale” with 15 items are acceptable and this scale can be used in the clinical environment. A further study within the nursing community is recommended to confirm the findings.

**Supplementary Information:**

The online version contains supplementary material available at 10.1186/s12912-024-01759-6.

## Introduction

In recent decades, demonstrations of sexual harassment (SH) and gender discrimination have infiltrated all professions, and healthcare workers are also facing this serious problem [1] SH is defined as repeated and unpleasant sexual behaviors common in the workplace including verbal, physical, psychological, and visual types, imposed on a person, regardless of their consent and are associated with humiliation, insult or threat to the health of the victims. This happens in a context where power relations are unequal [2]. According to the guidelines of Equal Employment Opportunity Commission (EEOC), SH includes: unwelcome sexual advances, requests for sexual favors, and other verbal and physical behavior of a sexual nature [3]. These actions are considered SH when: (1) Submission and absolute obedience is a condition of the person’s employment; (2) The acceptance or non-acceptance of such behaviors by the individual is a basis for making a career decision; (3) These behaviors interfere with a person’s work performance and turn the work environment into an intimidating, hostile, and insulting environment [4]. It should be noted that today part of SH is related to cyber sexual harassment (CSH). CSH includes conduct that meets the definition of SH but occurs via electronic communication technology and social networking sites [5].

Related studies show that SH is widespread in workplaces and has different prevalence in different countries. Healthcare workers, especially nurses, are more likely to be exposed to aggressive behaviors, such as SH [6, 7]. The prevalence of SH in emergency medical personnel in Korea has varied from 5.6% for men to 28.1% for women [8]. According to Budden’s study, 50.1% of Australian nursing students had experienced SH [9]. A review study showed that about a quarter of nurses around the world had been exposed to SH, indicating that the prevalence of SH of nurses in Asia was 21.6%, Europe 16.2%, the Middle East 22.4%, England 38.7% [10], China 3.9% [11] and Iranian nurses 1.07–9.5% [12].

Following the occurrence of SH behavior, there is a feeling of insecurity in the work environment, the working conditions become unstable and shaky, as a result, it causes psychological erosion and damages such as stress, fear, aggression, depression, and numerous physical problems which leads to confusion in work activities and family life [13, 14]; Therefore, it weakens the nurse’s ability to provide safe and competent care and inclines them toward issues such as resignation, frequent absences, reduced energy, reduced work efficiency, reduced creativity, incompatibility with colleagues, reduced professional satisfaction, reduced quality of patient care [15]. A large number of victims consider reporting the incident to be pointless, or due to previous experiences and even lack of knowledge of policies and guidelines, they prefer to remain silent and refuse to report [16].

To our best knowledge and literature review, it seems that there is no reliable instrument that can cover the issue in question; Because the instruments used to investigate SH, in various studies, are mostly researcher-made and without considering the experiences of the victims [17]. Fitzgerald et al. designed the Sexual Experiences Questionnaire (SEQ) in 1988 [18]. The results of a meta-analysis in 2007 showed that 59% of articles published in the field of SH at work used the SEQ [19]. However, Gutek et al. believed that the SEQ has weak psychometric properties and some disadvantages like its time frame, number of items, and wording of items. In addition, this questionnaire defines SH in a very general way and does not address the details clearly, and most importantly, it is not clear what definition of SH it evaluates [20]. Another instrument called the standard questionnaire of Workplace Violence (WPV) in the Health Sector designed in 2003 aimed to achieve information on the extent of workplace violence in the health sector from different geographical regions of the world [21]. WPV has been used repeatedly in studies of different countries [22, 23]. WPV is about the employees of the health department and it is not specifically addressed to nurses, and the main is “violence” and the category of SH has as a little part and its importance has been downplayed. In addition, WPV does not mention the examples of violence and only the frequency of violence in four physical, psychological, sexual, and racial dimensions is examined [21].

Even though sexual harassment is a significant issue among nurses, there have been very few studies on this topic, and nurses’ experiences in this area have not been explored in depth. As far as this is concerned, no valid and reliable scale is available in Persian. It is essential to develop an instrument that is valid and reliable to assess sexual harassment to identify the incidence of the behavior and develop prevention programs. Therefore, the purpose of the present study was to design and psychometrically measure the Nurses’ Sexual Harassment Scale (NSHS).

## Materials and methods

### Study design

This study is the second phase of a mixed method study. There were two general phases in this exploratory sequential mixed methods study: the development of items with the qualitative method, the literature review (inductive and descriptive), and the evaluation of the items’ psychometric properties. In the first phase, a qualitative approach with a conventional content analysis method was used to explain nurses’ SH experiences and discover their related topics. Moreover, in the second phase, a methodological research method was used to construct the Nurses Sexual Harassment Scale (NSHS) and validate it. In this research, Devellis’ scale development and the Classical Test Theory approach were used. The classical test theory is based on several assumptions: According to this theory, test scores reflect three general concepts: the observed score (O), the true score (T), and the error score (E). The premise of the classical theory is that each individual has a true score on the test, but due to measurement error, the researcher is unable to attain the true score. The observed score is the sum of the unobservable true score and the error score; O = T + E; the error score and the true score are assumed to be independent of each other; the average error score in the population of test takers is assumed to be zero [24]. A scale development process consists of eight stages: (1) Determine what you want to measure, (2) Generate an item pool, (3) Determine the format for measurement, (4) Have initial item pool reviewed by experts, (5) Consider inclusion of validation items, (6) Administer items to a development sample, (7) Evaluate the items, and (8) Optimize scale length [25]. The study was conducted in Kerman, the largest city in the southeast Iran, with a population of 712,000.

### Interviews and qualitative analysis

In this section, nurses and some faculty members from 8 public and private hospitals who had experienced sexual abuse were interviewed. Detailes for the qualitative part of the study was published in Zeighami et. al.’s article in 2022 [26].

### Items pool generation

Themes, categories, codes, and quotes from the results of qualitative data analysis [26] were used to design the pool of scale items. In addition to the qualitative findings, an electronic search of databases was conducted. EMBASE, PubMed, Web of Science, Scopus, Google scholar and Iranian article databases such as SID, MagIran were searched without time limit and with keywords of sexual harassment, questionnaire/scale/instrument, psychometrics, content analysis, qualitative study using combined search strategies.

### Content validity

For content validity, we conducted two qualitative and quantitative methods with 14 experts. In the qualitative content validity, 14 experts mentioned their opinions on content coverage, grammar, use of appropriate expressions, and appropriate location of items. To check content validity quantitatively, we checked content validity ratio (CVR) and content validity index (CVI). The content validity ratio (CVR) measures the necessity of an item on a three-point Likert scale (necessary - useful but not essential - not essential). Using the Lawshe table, a minimum content validity ratio was calculated [27, 28]. The number of experts in this study was 14, and the minimum acceptable content validity ratio was 0.51, so items with a CVR greater than 0.51 were kept. To determine CVI, Waltz & Bausell content validity index was used. they typically use a 4-point ordinal scale for item ratings. They considered 3- or 5-point rating scales but advocated for the use of a 4-point scale to avoid a neutral and ambivalent midpoint. While various labels for the four points along the item-rating continuum have been proposed in the literature, the one advocated by Waltz and Bausell, which includes 1 = not relevant, 2 = somewhat relevant, 3 = quite relevant, and 4 = highly relevant, appears to be commonly used. Subsequently, for each item, the I-CVI is calculated as the number of experts giving a rating of either 3 or 4 (thus dichotomizing the ordinal scale into relevant and not relevant), divided by the total number of experts [29]. The Item content validity index (I-CVI) and the scale content validity index (S-CVI) were calculated. The validity of items with I-CVI of 0.8 and S-CVI of 0.9 or more were acceptable [30].

### Face validity

Two qualitative and quantitative methods were used to determine face validity. In order to determine the qualitative face validity, 12 sexually harassed nurses were interviewed face-to-face and the level of difficulty, relevancy, and ambiguity of items were investigated. For quantitative face validity, the Item Impact Method was used to reduce and remove inappropriate items and determine the importance of each item. Items with the impact score of 1.5 or more remained for further analysis [31].

### Validation of items in a pilot sample

The initial scale was conducted in a pilot sample of 50 nurses using convenience sampling method. For Item Analysis, the percentage of responses to each response option of the items, missing value of each item, central tendency and dispersion indices, variability skewness (Pearson’s skewness index), and kurtosis indices, and the floor and ceiling effect were investigated [32]. Items with more than 15% missing values should be deleted, revised, or replaced [33]. It has also been shown that the number of missing values for an individual respondent should not exceed 20% [34]. The skewness of an item was determined by the absolute value of skewness being outside the range of ± 3 [35]. Also, the skewness values of the total score of the scale should not be less or more than − 1 and 1 [36]. If the absolute value of skewness is outside the range of ± 4, that item has kurtosis [37]. According to some sources, the kurtosis value of each item is acceptable up to the range of ± 8 [38]. If more than 80% of the samples have chosen the highest option or the lowest option in the Likert scale, the ceiling or floor effect is taken into account and the corresponding item is removed. Floor and ceiling effects are calculated for the entire instrument in addition to the items. If more than 80% of the samples get the maximum and minimum scores of the scale, the level of floor and ceiling effects is unacceptable [39]. Also, in this stage, the initial reliability of the tool was measured; In this way, the internal consistency of the tool was calculated with Cronbach’s alpha method. Also, the item analysis was done; For this purpose, Inter Item Correlation and Item Total Correlation were used. In this regard, the amount of alpha change in case of removing each item was investigated. It should be noted that if the Corrected Item Total Correlation was less than 0.3, the selected item was removed [40].

### Construct validity

After conducting the pilot study, the instrument should be implemented in a large sample representing the community in order to calculate the construct validity and reliability. In the current study, A total of 316 nurses were selected by convenience sampling to complete the scale. In the current study, structural validity and convergent validity were used to measure construct validity.

#### Structural validity

In order to determine structural validity, methods such as convergent and divergent correlation matrix of areas with each other and each question with its own area and also factor analysis were used [41]. Factor analysis includes exploratory and confirmatory analysis [40]. The method of factor analysis in this research was exploratory. In this stage, the number of samples was considered 10 times the number of items, and finally, 316 scales were completed by the nurses in the form of self-report [25]. In order to perform exploratory factor analysis, SPSS software version 27 was used. In the exploratory factor analysis, the Kaiser- Meyer-Olkin (KMO) statistic was calculated to check the adequacy of the sample, and the value of 0.8 or more is considered suitable [42]. While performing exploratory factor analysis, the type of rotation should be specified. Rotation is done to simplify and clarify the structure [43]. In the present study, according to the strength of correlation between the factors, the type of Varimax rotation was chosen. The minimum acceptable factor loading to maintain each item in the factor was considered to be 0.4. Factors are named based on the items of each factor after their extraction [39]. In order to determine the number of factors to be extracted, a decision was made based on the eigenvalue ≥ 1 and the scree plot [38]. Missing values should be managed during statistical analysis (43); For example, by replacing missing values with the mean or median or removing cases that have more than 20% missing data [44]. In the present study, missing values were replaced by the median.

#### Convergent validity

Convergent validity refers to the degree to which a measure is correlated with the measures or tasks that should tap the same construct [45]. In the present study, the Impact of Event Scale– Revised (IES-R) was used. 316 nurses completed both scales. The reason for choosing IES-R is that this scale evaluates post-traumatic stress syndrome (PTSD). Many studies have shown the relationship between the experience of sexual abuse and PTSD symptoms [46, 47]. On the other hand, due to the lack of a standard SH instrument based on Iranian culture, it was decided to use IES-R for convergent validity.

The Impact of Event Scale (IES) was developed by Horowitz et al. in 1979 and was the first PTSD diagnostic tool that was developed before the criteria for the diagnosis of PTSD were defined in DSM-III [48]. The revised version (IES-R) was designed in 1997 by Weiss and Marmar according to DSM-IV criteria. The IES-R is a self-report instrument that assesses psychological symptoms that occur after a specific traumatic event. The tool includes 22 items and consists of three subscales of intrusive thoughts, avoidance, and hyperarousal. Respondents complete the frequency of experiencing each symptom on a four-point Likert scale from 1 (never) to 4 (very much). The total score varies between 0 and 88. The cut-off point is 33, which shows the highest diagnostic accuracy. Therefore, people who score equal to or more than 33 are diagnosed with PTSD. Weiss and Marmar reported a good internal consistency and test-retest reliability, and its validity indices to be acceptable [49]. The Persian version of IES-R’s content validity was evaluated by the faculty members of Kerman University of Medical Sciences, and its reliability were evaluated with Cronbach’s alpha as 0.87 for the whole scale and 0.8, 0.79, and 0.73 for each subscale of intrusive thoughts, avoidance and hyperarousal, respectively. Therefore, the Persian version of IES-R shows acceptable reliability [50]. In the present study, IES-R’s Cronbach’s alpha was calculated for the whole scale as 0.94 and for each subscale of intrusive thoughts, avoidance, and hyperarousal were 0.87, 0.89, and 0.89, respectively.

### Reliability

Internal consistency, test-retest and McDonald’s Coefficient Omega were used to determine the reliability. In the present study, the internal consistency of the scale was measured on two steps; One before factor analysis with 50 samples, and one with 316 samples after factor analysis. Cronbach’s alpha coefficient higher than 0.9 is considered excellent, 0.7–0.9 is good, 0.5–0.7 is average, and less than 0.5 is unacceptable [51]. McDonald’s Omega is a reliability coefficient that is conceptually similar to Cronbach’s alpha coefficient and also takes the strength of the relationship between items into account [52]. In the present study, McDonald’s Coefficient Omega was calculated on the main sample (316 samples). Also, for the test-retest method, 30 nurses were asked to fill the scale on two occasions, with a time interval of two weeks; Then, Intra Class Correlation (ICC) was calculated for all dimensions as well as for the entire scale. If ICC is higher than 0.8, the reliability is favorable [51].

### Final item numbers of the scale

At this stage, the final scale was prepared. It should be kept in mind that shorter scales are more appropriate because they are more favorable to the respondents, on the other hand, longer scales are more reliable. It is obvious that maximizing one leads to decreasing the other [25]. In addition to the results of the previous steps, the ease of use or practicality of the scale was calculated by determining the percentage of unanswered questions. The time of the response was also studied on average from the samples and the final decision was made about the length of the scale.

## Results

### Qualitative part of the study

The results related to the qualitative part of the study, as well as the characteristics of the participants and the qualitative analysis of the results, are described in the study by Zeighami et al. [26]. According to the qualitative content analysis, 31 items were identified.

### Litrature review for item generation

Related studies, considering the purpose of the study and having the access to the full text of the articles, were searched; The most relevant articles were selected. A total of 35 articles were found, and no tools were found among articles conducted in Iran. 18 tools designed in other countries were explored to complete the items pool and modify some items [18, 53–69]. After reviewing the texts and tools available in the field of SH, 17 items were added to the scale and some items were revised. Therefore, at the end of the item compilation stage, there were 48 items: verbal sexual harassment (14 items), physical sexual harassment (11 items), visual sexual harassment (7 items), sexual deception (10 items), cyber sexual harassment ( 6 items).

### Response options

In the present study, a 5-point Likert scale (never, rarely, sometimes, often, always) was designed. Conducting a pilot study on 50 sexually harassed nurses showed that 7.78% of the responses were assigned to the middle answer option (sometimes). Therefore, the selection of a 5-point Likert scale (with a middle answer option) for the present scale was unimpeded.

### Content validity

#### Qualitative content validity

14 experts [a master’s degree in nursing, a doctorate in clinical psychology, a doctorate in counseling (with an experience of more than 8 years of providing sexual counseling), and 11 with a doctorate in nursing] reviewed the scale. According to experts’ opinions, some items were merged due to overlapping and similar concepts, 10 items (items 1, 5, 14, 17, 32, 35, 39, 40, 41, 46) were removed for reasons such as vagueness, similarity or having a overlap with other items. Also, the item “winking” was added. According to experts, the NSHS had sufficient comprehensiveness (Appendix 1).

#### Quantitative content validity

The same 14 experts participated to determine CVR and CVI. Based on the results of the CVR, only one item (item 37) had a coefficient less than 0.51, which was a candidate for removal from the scale. The results showed that the CVI of all items were higher than 0.8 (between 0.85 and 1). In only one item, the CVI was less than 0.9. Also, the CVI of the entire scale was 0.982. After removing eleven items, the CVI of the entire scale increased to 0.989. In the end, eleven items out of 48 items were removed. As a result, at the end of this stage, the scale contained 37 items (Appendix 1).

### Face validity

#### Qualitative face validity

12 sexually harassed nurses were interviewed face-to-face. The participants recognized all the items as appropriate, and there was no need to change any of the items.

#### Quantitative face validity

The scale was provided to 12 sexually harassed nurses. According to the results, The item impact score of 22 items was a full score of 5. The “intentional jostling” item had the lowest item impact score of 4.16. At this stage, no items were removed from the scale and the final scale with 37 items was prepared for the pilot study (Appendix 1).

### Item analysis (pilot test)

After determining the face and content validity, 50 nurses filled out the scale. The average age of the pilot sample was 31.66 years with a minimum of 23 and a maximum of 47 years. Their working experience was 6.83 years with a minimum of one and a maximum of 18 years. The majority of the participants were married (50.9%), had a bachelor’s degree (75. 5%), with a nurse position (90.6%), and rotating shifts (90.6%). 83% of them used social networks, spent an average of 156 min of their time daily. At this stage, all the items that had floor and ceiling effects of more than 80%, Corrected Item-Total Correlation of less than 0.3, Inter Item Correlation of more than 0.7, skewness of ± 3 and kurtosis of ± 4 or more were determined, and according to the opinion of the research team, some of these items were removed and some were kept (Table 1).

**Table 1:** Results of item analysis based on a pilot study on 50 nurses (pilot test)

Exclusion criterion	< 0.3	-	80% <	≤ -3 to + 3 ≤	≤ -4 to + 4 ≤	Changes
Items	Corrected Item-Total Correlation	Cronbach’s alpha in case of item deletion	Floor Effect	Skewness	Kurtosis	
1. Sending you text messages with sexual content	0.655	0.952	80%	2.261	4.407	Remained with the opinion of the research team
2. Sending or showing you vulgar photos and videos and links through social networks and email	0.562	0.953	84%	2.588	5.836	Remained with the opinion of the research team
3. Threats to publish photos, videos, and private chats if there is no sexual willingness	0.251	0.954	98%	7.071	50	Remained
4. Asking you to send a nude photo of a part your body	0.386	0.954	92%	3.96	16.477	Remained
5. Sending you a nude photo of their body	0.451	0.953	96%	6.21	40.203	Remained
6. Expressing of affection and romantic words to attract sexual willingness.	0.803	0.951	92%	1.194	0.236	Remained with the opinion of the research team
7. Good behavior to attract sexual willingness.	0.802	0.950	60%	1.062	0.198	Remained
8. False promise of marriage to attract sexual willingness	0.733	0.951	78%	2.427	5.565	Remained with the opinion of the research team
9. Forced to establish an unusual relationship to maintain working conditions.	0.494	0.953	92%	2.468	5.162	Remained with the opinion of the research team
10. A tempting financial or professional offer in exchange for sexual willingness	0.638	0.953	90%	3.450	12.378	Remained
11. Sexually teasing and dirty jokes.	0.848	0.950	54%	1.027	-0.87	Remained
12. Deliberate interpretation of your normal words into sexually charged words (verbs like “do” and words like “thing”…)	0.810	0.950	58%	1.464	1.514	Remained
13. Insisting to have your contact number	0.791	0.951	56%	1.288	0.680	Remained
14. Giving you a contact number, with the intention of establishing an informal friendship	0.795	0.951	58%	1.609	2.392	Remained
15. Insist on meeting outside of work	0.755	0.951	56%	0.981	-0.282	Remained
16. Suggesting a temporary marriage to satisfy lust and have sex.	0.285	0.954	94%	3.821	13.124	Remained
17. Expressing unusual admiration of your style and appearance	0.830	0.950	66%	1.194	-0.017	Remained
18. Expressing unusual admiration of your body.	0.823	0.950	62%	1.293	0.278	Remained
19. Talking openly about sexual matters.	0.737	0.951	60%	1.168	0.365	Remained
20. Telling stories with sexual content.	0.499	0.953	74%	1.727	1.886	Remained
21. Addressing you with sexual insults	0.266	0.955	84%	3.164	10.365	Remained
22. Lustful stares	0.800	0.951	36%	0.599	-0.830	Remained
23. Exposing their sexual organs	0.144	0.954	94%	3.821	13.124	Removed
24. Touching their sexual organs in front of you	0.235	0.955	88%	3.717	14.065	Remained
25. Showing sexual symbols (for example, showing some sexual acts with hands)	0.642	0.952	82%	2.983	10.053	Remained with the opinion of the research team
26. Sending air kisses from a distance	0.506	0.953	76%	1.805	2.514	Remained
27. Winking	0.791	0.951	50%	0.691	-0.798	Remained
28. Touching your body	0.483	0.953	78%	1.858	2.082	Remained
29. Intentional jostling.	0.830	0.950	60%	0.943	-0.546	Remained
30. Kissing	-0.056	0.955	98%	7.071	50	Removed
31. Hugging	-	-	100%	-	-	Removed
32. Standing too close to you in an unusual way	0.823	0.950	46%	1.063	0.274	Remained
33. Making a contact of their sexual organs with your body.	0.494	0.953	86%	2.721	7.353	Remained with the opinion of the research team
34. Touching your sexual organs	0.384	0.954	94%	3.821	13.124	Remained
35. Forcing you to touch their sexual organs.	-	-	100%	-	-	Removed
36. Removing your clothes (headcover, uniform,…) by force.	-	-	100%	-	-	Removed
37. Raping.	-	-	100%	-	-	Removed

### Reliability: internal consistency (pilot test)

Cronbach’s alpha of the whole scale with 37 items was 0.95. After removing 6 items, Cronbach’s alpha coefficient reached 0.956. Finally, the scale with 31 items was prepared for the construct validity.

### Construct validity

In the current study, structural validity and convergent validity were used to measure construct validity.

#### Structural validity

A total of 316 nurses working in hospitals affiliated to Kerman University of Medical Sciences participated in this stage of the study and completed the scale. Since the scales with 20% or more missing data sould be removed [34], none of them were removed, and finally 316 scales were entered item analysis and factor analysis.

The average age of the participants in this part of the research was 32.6 years. The average working experience of the personnel was 8.74 years. 59% of the samples were married, 81.2% had undergraduate education, and 79.1% had experienced some types of SH. 90.07% of nurses with harrasment experience were abused by someone of the oposite sex. 96.5% of the samples used social networks, spending an average of 185 min a day using it. The most used social network was WhatsApp with 42.81% (Table 2).

**Table 2:** Demographic characteristics of the nurses participating in the research (*N* = 316)

Quantitative Variables	Mean ± Standard Deviation
Age (years)	32.6 ± 6.802
Work experience (years)	8.74 ± 6.409
Hours spent on social networks during the day and night (minutes)	185.4 ± 128.82
**Qualitative variables**	Frequency* (%**)
**Marital Status**	
Single	115 (36.5)
Married	186 (59)
Divorced	11 (3.5)
Widow(er)	3 (1)
**Education**	
B.Sc.	255 (81. 2)
M.Sc.	59 (18.8)
**Position**	
Supervisor	5 (1.6)
Head Nurse	9 (2.8)
Nurse	302 (95.6)
**Shifts**	
Fixed	24 (7.6)
In Circulation	292 (92.4)
**Hospitals**	
A	112 (43.6)
B	53 (20.6)
C	69 (26.8)
D	23 (8.9)
**Wards**	
ICU	62 (22.3)
Emergency Room	66 (23.7)
Dialysis	7 (2.5)
Psychiatry	24 (8.6)
Oncology	4 (1.4)
Internal	72 (25.9)
CCU	6 (2.2)
Surgery	14 (5)
Orthopedics	4 (1.4)
Pediatrics	4 (1.4)
Supervisory Office	4 (1.4)
Operation Room	9 (3.2)
Neurology	1 (0.4)
Eye	1 (0.4)
Offender’s Sex***	
Same Sex	7 (6.5)
Opposite Sex	97 (90.07)
Both Sexes	3 (2.8)
Offender’s Position***	
Doctor	41 (19.5)
Nurse	38 (17.75)
Office Clerk	30 (14.02)
Patient	37 (17.3)
Patient’s Companion	46 (21.5)
Other	22 (10.28)
**Use of Social Networks**	
Yes	304 (96.5)
No	11 (3.5)
Type of social network****	
Telegram	138 (20.44)
WhatsApp	289 (42.81)
Instagram	236 (34.96)
Twitter	9 (1.34)
Other	3 (0.45)

*Variable that are less than 316 in total are due to missing data

**Valid percent

*** Some participants were harassed by more than one gender and more than one position

**** Some participants used multiple social networks

#### Distributional items analysis

At this stage, the items that had ceiling and floor effects of more than 80%, skewness of ± 3 and kurtosis of ± 4 or more were identified. None of the items had a ceiling effect. Since the research topic is a taboo and due to the special cultural environment of Iran, some types of SH happen less often, hence there was a floor effect in 12 items. 7 items had skewness and 17 items had kurtosis effect. These items remained for factor analysis with the opinion of the research team.

#### Exploratory factor analysis

Before starting the factor analysis, the missing data of each item were replaced with the median of that item. It should be noted that the factor analysis was performed both by removing problematic items in the item analysis stage and without removing them. Then, different methods of factor extraction and rotation type were used and the results were compared (Table 3).

**Table 3:** General results of factor analysis with different methods

Number	Method	Rotation	Explained Variance	Kaiser-Meyer-Olkin (KMO)	Bartlett Test	Number of Dimensions	Number of Items	The number of items in each dimension	Variance of each Dimension
1	Principal Axis Factoring	Varimax	68.40	0.935	χ^2^ = 3636.11 Df = 105,105 *p* < 0.0001	2	15	9	56.7
								6	11.69
2	Principal Axis Factoring	Varimax	65.82	0.934	χ^2^ = 3703.93 Df = 120 *p* < 0.0001	2	16	9	54.06
								7	11.76
3	Principal Axis Factoring	Promax	67.48	0.943	χ^2^ = 5331.79 Df = 231 *p* < 0.0001	3	22	10	52.26
								6	8.93
								6	6.30
4	Maximum Likelihood	Varimax	67.08	0.939	χ^2^ = 4964.12 Df = 210 *p* < 0.0001	3	21	10	51.47
								6	9.88
								5	5.73
5	Maximum Likelihood	Promax	65.09	0.946	χ^2^ = 6120.60 Df = 300 *p* < 0.0001	3	25	13	51.37
								6	607.8
								6	5.11
6	Principal Axis Factoring	Promax	65.59	0.948	χ^2^ = 4629.18 Df = 153 *p* < 0.0001	2	18	10	58.87
								8	6.73
7	Maximum Likelihood	Promax	65.59	0.948	χ^2^ = 4629.18 Df = 153 *p* < 0.0001	2	18	8	58.87
								10	6.73

The results related to this method are reported considering the better and more meaningful factor analysis result without removing problematic items and considering the interpretability and better placement of items with the method of Principal Axis Factoring (PAF) and Varimax Rotation.

Two suitable factors were considered for this scale. These 2 factors explained a total of 68.4% of the total variance. The first factor is “latent sexual harassment” (CSH) (9 items) and the second one is “manifest sexual harassment” (OSH) (6 items). At the end of the exploratory factor analysis stage, by removing 16 items, the number of items reached to 15 (Table 4).

**Table 4:** Factors extracted from exploratory factor analysis using principal axis factoring method and varimax rotation

Items		Factor Load		Communalities	
		Factor 1	Factor 2	Extraction	Initial
1.	Insisting to have your contact number	0.835		0.735	0.73
2.	Giving you a contact number, with the intention of establishing an informal friendship	0.824		0.75	0.762
3.	Insist on meeting outside of work	0.809		0.707	0.71
4.	Good behavior to attract sexual willingness.	0.756		0.697	0.721
5.	Expressing unusual admiration of your style and appearance	0.746		0.657	0.713
6.	Expressing of affection and romantic words to attract sexual willingness.	0.742		0.681	0.709
7.	Expressing unusual admiration of your body.	0.732		0.687	0.735
8.	Standing too close to you in an unusual way	0.661		0.568	0.611
9.	Lustful stares	0.637		0.509	0.556
10.	Showing sexual symbols (for example, showing some sexual acts with hands)		0.831	0.744	0.671
11.	Making a contact of their sexual organs with your body.		0.748	0.654	0.627
12.	Touching their sexual organs in front of you		0.731	0.58	0.537
13.	Touching your body		0.683	0.572	0.583
14.	Sending air kisses from a distance		0.665	0.588	0.597
15.	Addressing you with sexual insults		0.599	0.425	0.416
**Eigen value**		8.505	1.755		
**Explained variance**		56.7	11.698		
**Cumulative variance**		68.399			

#### Convergent validity

For convergent validity, all nurses who completed the scale in the construct validity section also completed the IES-R, but due to the fact that some scales had missing values, 303 completed scales were used for convergent validity (response rate = 95.9%). Convergent validity is confirmed by a Spearman correlation coefficient greater than 0.4 [70]. According to this study, the Spearman correlation coefficient between IES-R and latent sexual harassment subscales was 0.671. The correlation coefficient with manifest sexual harassment subscales was 0.423. The correlation between the total score of these two scales was 0.668, indicating a strong correlation; thus, the convergent validity was confirmed (Table 5).

**Table 5:** Correlation between the subscales and the total scale of the nurses sexual harassment scale with the the impact of event scale - revised (*N* = 303)

The Sexual Harassment in Nurses Scale	The Impact of Event Scale - Revised	
	***P*** value	r
Latent Sexual Harassment	< 0.0001	0.671
Manifest Sexual Harassment	< 0.0001	0.423
The Total Scale	< 0.0001	0.668

### Reliability

Cronbach’s alpha and McDonald’s omega reliability coefficient were controlled in a sample size of 316 samples. The Cronbach’s alpha of the scale with 15 items was 0.944. Also, the Cronbach’s alpha for the latent and manifest sexual harassment subscales were 0.944 and 0.893, respectevely. The omega coefficient of the scale with 15 items was 0.945. Also, the omega coefficient for the latent and manifest sexual harassment subscales were 0.943 and 0.894, respectevely. The test-retest method was performed to evaluate the stability of the scale. Therefore, 30 nurses were asked to complete the final edition of the scale two times with two weeks apart, and then the the intra-class correlation coefficient for all dimensions were calculated as well as for the entire scale (Table 6).

**Table 6:** The cronbach’s alpha, McDonald’s omega, and intra-class correlation coefficients of subscales and the total scale of the sexual harassment in nurses scale

Dimensions (Subscales)	Cronbach’s alpha correlation coefficient	McDonald’s omega correlation coefficient	Intra-class correlation coefficient	Confidence Interval	
Latent Sexual Harassment	0.944	0.943	0.888	0.534	0.959
Manifest Sexual Harassment	0.893	0.894	0.953	0.9	0.978
The Total Scale	0.944	0.945	0.917	0.665	0.969

### Practicality

The practicality or ease of use of the scale was calculated by determining the percentage of unanswered questions which ranged from 0 to 0.6%. Also, in average, 0.081of the scale questions were not answered. The average response time to the scale was 4 min and 20 s with minimum of 2 min and 30 s and a maximum of 12 min.

### Final edition of the scale and scoring

The Sexual Harassment in Nurses Scale has 15 items in two subscales of “latent sexual harassment” (9 items) and “manifest sexual harassment” (6 items). The response range of the scale includes never = 0, rarely = 1, sometimes = 2, often = 3, and always = 4. There are no inverse items in the scale. The minimum score of the scale is zero and the maximum score is 60, and a score of zero indicates no experience of sexual harassment, and as the score increases, it indicates more experiences of sexual harassment.

## Discussion

This study led to the developing of the first Nurse Sexual Harassment Scale in Iran. Based on the results of this research, a scale with 15 items was created. The scale has two dimensions: “hidden sexual harassment” (9 items) and “manifest sexual harassment” (6 items). This scale is scored on a 5-point Likert scale. The Nurse Sexual Harassment Scale showed a good reliability and construct validity rating. Instruments and scales can be evaluated based on their measurement properties. Based on similar studies, some structures have been discussed regarding this field. A summary of these scales’ psychometric characteristics can be found in Table 7. One of these instruments in the field of SH is the Sexual Experiences Questionnaire (SEQ) which was developed by Fitzgerald et al. in 1988 in the United States and is one of the first tools in this field. SEQ has 28 items and five factors as gender harassment (7 items), seductive behavior (5 items), sexual bribery (4 items), sexual coercion (4 items), and sexual assault (7 items) and a criterion item (I have been sexually harassed) [18]. SEQ uses a 3-point Likert spectrum including never, once, and more than once, while NSHS uses a five-point Likert scale for answering. In SEQ, confirmatory factor analysis was used for construct validity, while in the present study, exploratory factor analysis was used to extract factors. Both studies showed good psychometric characteristics for both instruments. Furthermore, there are similarities between NSHS and SEQ. For example, SEQ in the first subscale of sexual harassment the item of “I have been repeatedly and uncomfortably exposed to the stares,…” is similar with NSHS item “Lustful stares”. However, there are also some differences between these two scales. For example, NSHS mainly includes hidden sexual harassment in which the harasser attempts to entice and gain the victim’s attention and cooperation in some way. At the same time, SEQ contains sexual coercion, in which the harassed individual is forced to comply with the harassment to retain their employment position. These differences may be attributed to cultural differences between the two environments.

The second version of SEQ was examined in workers population and it was published in 1995 under the name of Sexual Experiences Questionnaire-Workers Version (SEQ-W). SEQ-W considers SH to include three factors: gender harassment (5 items), unwanted sexual attention (7 items), and sexual coercion (5 items) with a total of 17 items. However, the number of factors in NSHS is 2 with 15 items. SEQ-W, like NSHS, is evaluated on a 5-point Likert scale from never [1] to always [5]. According to the results of both studies, the reliability coefficients of NSHS are higher. Unlike the present study, which used exploratory factor analysis for construct validity, for SEQ-W, confirmatory factor analysis was used to extract factors [18]. Some items of NSHS are the same and similar to SEQ-W’s. For example, in SEQ-W subscale of SH, the item “made offensive remarks” with NSHS item “addressing you with sexual insults " are similar. Although the aforementioned instruments are conceptually similar to NSHS and share some items, but in NSHS, the items have been adjusted in such a way as to be consistent with the specific culture of Iran. On the other hand, those instruments are used in general and in different work environments, while NSHS is specific and was developed only to investigate sexual harassment in nurses [18, 20, 53].

The Sexual Experiences Questionnaire-Department of Defense (SEQ-DoD) was designed to assess sexual harassment in the US military by Fitzgerald et al. in 1999. The SEQ-DoD has 23 items in four dimensions. Its dimensions includes gender harassment (sexist hostility) with 4 items, gender harassment (sexual hostility) with 8 items, sexual coercion with 5 items, and unwanted sexual attention with 6 items. This instrument evaluates responses on a 5-point Likert scale ranging from never [1] to most of the time [5]. The shortened form of the questionnaire (SEQ-DoD-S) was prepared in 2002 and has 16 items and the same four factors with each factor having 4 items. The shortened form maintains the appropriate psychometric properties and has the same performance as the original questionnaire [55, 59]. In this study, exploratory factor analysis was used for the construct validity of the scale, while both questionnaires SEQ-DoD and SEQ-DoD-S used confirmatory factor analysis. Both of these questionnaires, like NSHS, have a good reliability coefficient. Comparing the factors and items of SEQ-DoD with NSHS, it can be said that despite the greater number and the different naming of factors in SEQ-DoD, the content of some items are common with NSHS. SEQ-DoD and SEQ-DoD-S were designed for use in the US Army, while NSHS was specifically designed to measure SH in nurses who are part of the healthcare system. While the NSHS primarily covers hidden sexual harassment, the SEQ-DoD and SEQ-DoD cover even more severe harassment, such as coercion and sexual assault.

The Sexual Experiences Questionnaire-Latin version (SEQ-L) was adapted in 2001 by Cortina based on the revision of SEQ. SEQ-L examines the prevalence of sexual harassment among working Latinos in the United States, especially working-class Mexican American women with limited education and relatively low acculturation. This instrument has 20 items in three dimensions. The first component is sexist hostility (4 items), the second component is sexual hostility (4 items), and the third component is unwanted sexual attention (12 items). It meseaures sexual harassment on a 5-point Likert scale (from never to most of the time) [61]. Comparing two instruments, SEQ-L has more factors and items. NSHS is similar to SEQ-L in the sexist hostility component in one item, the sexual hostility component in one item, and the unwanted sexual attention component in 7 items in terms of content. The type of factor analysis is different in two instruments, but both have good reliability coefficients. SEQ-L was designed to be used in Latin culture and for workers with low literacy level, in most cases of sexual harassment, obscene words, and sexual insults were used. While the NSHS is specifically designed to measure sexual harassment among Iranian nurses, such insults and sexually offensive language are uncommon in nursing environments.

Another instrument in this field is the Sexual Experiences Survey (SES), which was developed in 1982 by Koss & Oros in the United States. This tool is a self-report instrument of coercive and aggressive sexual experiences designed to classify women and men based on different degrees of sexual assault and victimization and is able to identify hidden rape victims. The initial form of SES contains 13 yes-no questions that explicitly refer to sexual relations with varying degrees of coercion, threat, and force. Factor analysis showed that this instrument contains one factor [57, 58]. SES was revised in 1985 by the original authors to increase clarity, improve consistency with the statutory definition of rape, and reflect greater degrees of sexual assault and victimization. The latter form contains 10 yes-no questions [57]. Comparing NSHS with SES, it can be said that this survey is almost completely different from NSHS both in terms of the number of dimensions and items. SES has only one factor and only investigates rape and sexual intercourse by resorting to different degrees of violence and force, while NSHS study is more comprehensive which does not consider sexual harassment only in being a victim of rape and has considered different levels for sexual harassment and classified it in two factors of latent and manifest sexual harassment. Both instruments have used exploratory factor analysis to extract factors, and Cronbach’s alpha coefficient is desirable for both instruments. Also, NSHS is specific and examines sexual harassment in nurses, who are a huge part of the medical staff.

The Sexual Harassment Inventory (SHI) was developed in 1998 by Murdoch & McGovern for use in the US military. This inventory has 20 items and three factors. The factors include 10 items of hostile environment, 6 items of quid pro quo (improvement of working conditions in exchange for sexual cooperation) and 4 items of criminal sexual misconduct, and the answers to the items are yes and no [60]. Comparing the factors and items of SHI with the NSHS, it can be said that the number and name of factors of SHI are different. Like other tools, there are similarities between these two instruments in terms of items. In SHI, confirmatory factor analysis was used for construct validity, and exploratory factor analysis was used in the present study. Both instruments have good reliability coefficients. SHI was designed for use in the US Army, although according to the opinion of the designers it can be adapted to different job groups, but NSHS was specifically designed to measure sexual harassment in nurses.

Comparing the above scales with the present study’s scale shows that although they have different dimensions and items, sexual harassment usually involves verbal, physical, visual, and psychological behaviors that are common in most societies. Regardless, since people experience and understand unpleasant feelings differently, scales appropriate to the culture of each group and society are needed. To create a culturally relevant measurement tool for sexual harassment, examining this concept within the cultural system of societies is necessary. It is possible to accomplish this goal using qualitative data collection methods, enabling nurses to describe their realities in their own words. These tools help identify the extent and nature of sexual harassment to prevent it from occurring. Due to this, the current research aims to develop a tool appropriate for Iranian society, particularly for nurses in their workplace. It will be effective to reflect the type and severity of sexual harassment of nurses to the authorities in order to plan for its prevention. This will be effective in maintaining the physical and mental health of nurses, as a result, it will prevent the reduction of the quality of patient care and leaving the job.

Like all other studies, we faced some limitations. For instance, although the interview was conducted individually and the nurses were assured of the confidentiality of the information and findings of the interview, however, due to the cultural sensitivity of SH in Iran, the participants may not have revealed all the sensitive information on this issue. Due to the fact that most of the participants in this study were women, caution should be taken in generalizing the results to both sexes. The findings of the present study were conducted in the southeastern part of Iran. Considering the many cultural and ethnic differences in Iran, these differences should be taken into account in future studies. However, according to the qualitative phase, we found some specific kinds of verbal SH and also another kind of physical SH which seemed to be more related to the nursing workplace. However, during the different phases of the study, these specific items were deleted or revised according to the expert opinions. Since numerous research literatures in different parts of the world have been used in this study, we suggest that our findings can be beyond the cultural context of Iran. IES-R was used to check the convergent validity. This is a PTSD-focused tool. PTSD may appears after more severe forms of sexual violence, therefore, it is sugessted to check convergent validity with other related concepts in the future studies. Although the present tool was designed and psychometrically tested in the group of nurses, according to the extracted items, it can be used in other groups as well. However, more research on better understanding of sexual harassment and its negative consequences in nurses seems necessary.

**Table 7:** Comparison of sexual harassment instruments in different studies

Instrument		Author(s)	Year	Sample size	Cronbach’s alpha reliability	Cronbach’s alpha range	Test-retest reliability	Number of items	Number of factors	Likert spectrum	Type of factor analysis
1.	SEQ	Fitzgerald et al.	1988	1,700 American students	0.92	0.62–0.86	0.86	28	5	3	Confirmatory
2.	SEQ-W	Fitzgerald et al.	1995	448 American West Coast Public Service Company workers	Not reported	0.42–0.85	-	17	3	5	Confirmatory
3.	SEQ-DoD	Fitzgerald et al.	1999	22,399 women working in the US military 5,855 men serving in the US Army	Women 0.94 Men 0.94	Women 0.83–0.94 Men 0.78–0.96	-	23	4	5	Confirmatory
4.	SEQ-DoD-S	Stark et al.	2002	22,035 women working in the US military 5,904 men serving in the US Army	Women 0.92 Men 0.91	Women 0.83–0.92 Men 0.78–0.94	-	16	4	5	Confirmatory
5.	SES	Koss & Oross	1985	448 psychology students of Kent University, Ohio, USA	Women 0.74 Men 0.89	-	0.93	10	1	yes-no	Exploratory
6.	SHI	Murdoch& Mc Goven	1998	448 female soldiers of the state of Minneapolis, USA	0.92	0.86–0.89	-	20	3	yes-no	Confirmatory
7.	SEQ-L	Cortina	2001	476 Latino people in adult schools or educational centers in San Diego, Chicago, USA	0.96	0.88–0.95	-	20	3	5	Confirmatory
8.	NSHS	Zeighami et al.	2021	316 nurses working in hospitals in Kerman, Iran	0.944	0.89–0.94	0.92	15	2	5	Exploratory

## Conclusion

Sexual harassment exists everywhere in the world and is not limited to borders, culture, nationality, religion, profession, and specific population, but its meaning and experience is a matter that is basically subjective and according to different cultural and socio-economic contexts has different meanings. Therefore, it is better to measure sexual harassment in each society with its own instruments. Therefore, the current research sought to construct and validate NSHS, in order to help identify the extent and dimensions of this social problem by deeply examining this concept in this population and creating related instrument. The findings of this study showed that sexual harassment consists of two components: latent sexual harassment and manifest sexual harassment. The important thing to consider is that nurses were the focus of compiling the items of this scale, in addition, with a brief review of related texts and instruments in the field of sexual harassment, an attempt was made to cover almost all aspects of sexual harassment. Therefore, we can hope that the scale that is the result of this in-depth study, considering that it was designed and psychometrically evaluated in the community of nurses, can even measure sexual harassment in the healthcare envirement. As the results of the current study showed NSHS is a valid and reliable scale to find and measure sexual harassment in healthcare envirement, especiallu in nurses.

### Electronic supplementary material

Below is the link to the electronic supplementary material.

Supplementary Material 1

## Data Availability

The datasets used for the current study are available from the corresponding author upon request.
