# Supplementary material for: Development and validation of the nurses sexual harassment scale in Iran
Source: BMC Nurs. 2024 Feb 8;23:107. doi: 10.1186/s12912-024-01759-6 (PMC10851457; doi:10.1186/s12912-024-01759-6)
Supplement: Supplementary file 1 — Supplementary Material 1 [file 12912_2024_1759_MOESM1_ESM.docx]

**Appendix 1: The results of content and face validity of sexual harassment in nurses scale**

| **Subscales and Items** | **Experts' reviews and suggestions** | **Content Validity Ratio (CVR)** | **Content Validity Index (CVI)** | **Final Item** | **Item Impact Score** |
| --- | --- | --- | --- | --- | --- |
| **Verbal Sexual Harassment** |  |  |  |  |  |
| 1. Sexual irony. | It is vague.  It overlaps with item 2.  Irony does not specify the meaning. | 0.71 | - | It was removed. | - |
| 2. Sexually teasing and dirty jokes. | - | 0.71 | 1 | It remained unchanged | 5 |
| 3. Interpreting ordinary words into sexually charged words (verbs like “do”). | This interpretation is from the one who is being abused, not the one who is doing the abuse.  Who interprets this? | 0.57 | 0.92 | Deliberate interpretation of your normal words into sexually charged words (verbs like “do” and words like “thing” ...) | 4.83 |
| 4. Insisting to have your contact number with the intention of a informal contact | There is no need for the last part "with the intention of a non-conventional relationship". Because the word insisting is a qualifier. | 0.71 | 1 | Insisting to have your contact number | 5 |
| 5. Insisting on an informal contact and friendship. | It is similar to items 4 and 6.  It overlaps with item 4. | 0.71 | - | It was removed. | - |
| 6. Giving you a contact number, with the intention of establishing an informal friendship | - | 0.71 | 1 | It remained unchanged | 5 |
| 7. Frequent requests to meet and have drinks or dinner with the intention of an informal contact | Using the word “insist” is better than “request”.  Frequent requests to meet outside of work | 0.71 | 1 | Insist on meeting outside of work | 5 |
| 8. Suggesting a temporary marriage to satisfy lust and have sex. | “Temporary marriage proposal” is sufficient and conveys the meaning. Temporary marriage is always for sex and everyone knows that, so it doesn't need to be explained further. | 0.57 | 0.92 | It remained unchanged with the opinion of the research team. | 5 |
| 9. Expressing unusual admiration of your clothes and appearance. | Items 9 and 10 are similar and can be merged.  Expressing unusual admiration of your clothes, style and appearance | 1 | 1 | Expressing unusual admiration of your style and appearance | 4.83 |
| 10. Expressing unusual admiration of your body. | It overlaps with item 9. | 0.85 | 1 | It remained unchanged with the opinion of the research team. | 5 |
| 11. Talking openly about sexual matters. | - | 0.85 | 1 | It remained unchanged. | 5 |
| 12. Telling sexual stories. | It overlaps with item 11  Telling stories with sexual content | 0.85 | 1 | Telling stories with sexual content. | 4.91 |
| 13. Swear words and sexual insults | It remained with minor changes in the tool | 0.85 | 1 | Addressing you with sexual insults. | 5 |
| 14. Addressing with nicknames such as whore or bitch. | It overlaps with item 13, I think it should be deleted.  “You” should be added after addressing. | 0.71 | - | It was removed with the opinion of the research team. | - |
| **Physical sexual harassment** |  |  |  |  |  |
| 15. Touching your body | Items 15, 17 and 22 are close to each other.  In my opinion, it should be written in two items and as follows:  Touching your genitals.  Touching or dabbing other parts of your body.  15, 16 and 17 can be merged | 1 | 1 | It remained unchanged with the opinion of the research team. | 4.91 |
| 16. Intentional jostling. | - | 0.85 | 1 | It remained unchanged. | 4.16 |
| 17. Hitting your leg or other part of your body | 15, 16 and 17 can be merged | 0.71 | - | It was removed with the opinion of the research team. | - |
| 18. Kissing. | - | 0.85 | 1 | It remained unchanged. | 4.83 |
| 19. Hugging. | Add “with force”. Sexual harassment is considered. | 0.85 | 1 | It remained unchanged with the opinion of the research team. | 4.32 |
| 20. Standing too close to you in an unusual way | - | 1 | 1 | It remained unchanged. | 4.83 |
| 21. Rubbing their genitals against your body | Sexual organs include genitals, breasts, hips and lips, so you should say sexual, not genital.  The contact of sexual organs with your body | 0.85 | 1 | Making a contact of their sexual organs with your body. | 4.32 |
| 22. Touching your genitals | Touching your sexual organs | 0.85 | 1 | Touching your sexual organs | 4.39 |
| 23. Forcing you to touch their genitals | Forcing you to touch their sexual organs. | 0.71 | 0.92 | Forcing you to touch their sexual organs. | 5 |
| 24. Removing your clothes (headcover, uniform, ...) by force. | - | 0.85 | 1 | It remained unchanged. | 5 |
| 25. Raping. | - | 0.85 | 1 | It remained unchanged. | 5 |
| **Visual sexual harassment** |  |  |  |  |  |
| 26. Lustful stares | - | 1 | 1 | It remained unchanged. | 5 |
| 27. Exposing their sexual organs | - | 0.85 | 1 | It remained unchanged. | 5 |
| 28. Stimulating their own genitals in front of you | Touching their sexual organs in front of you | 0.85 | 1 | Touching their sexual organs in front of you | 5 |
| 29. Showing sexual symbols | It is vague.  Give an example | 0.85 | 1 | Showing sexual symbols (for example, showing some sexual acts with hands) | 5 |
| 30. Sending kisses from afar | Consider the annoyingness of the act. | 0.85 | 1 | Sending air kisses from a distance | 5 |
| 31. Peeking and observing you in an unusual way | I think this item is very subtle. Be deleted.  It overlaps with item 26.  It is better to add a winking item instead of this item | 0.71 | 0.92 | Winking | 4.91 |
| 32. Repeatedly and unusually putting themselves at your sight | The way of writing and expression is different from previous items.  Being frequently and unusually at your sight. | 0.85 | - | It was removed. | - |
| **Seduction** |  |  |  |  |  |
| 33. Expressing of affection and romantic words with sexual goals. | Instead of the word "sexual" write the word "unusual", whoever has filled out the questionnaire so far will understand the meaning.  Expression of affection and romantic words to attract sexual willingness. | 1 | 1 | Expressing of affection and romantic words to attract sexual willingness. | 4.91 |
| 34. Good service to attract sexual willingness. | It is better to use other words for clarification  It seems better to “have sex” than “sexual willingness”.  It is not clear. | 1 | 1 | Good behavior to attract sexual willingness. | 4.91 |
| 35. Offering a bribe to get sexual willingness | I think, the name of this work is not "bribery".  Financial offer to attract sexual willingness | 0.85 | - | It was removed with the opinion of the research team. | - |
| 36. Sexual abuse with false promise of marriage | False promise of marriage to attract sexual willingness. | 1 | 1 | False promise of marriage to attract sexual willingness | 5 |
| 37. Forced to wear special clothing, make-up, nail polish, etc. by the work environment in order to be attractive and seductive | It is not clear at all.  it is vague.  It is ambiguous and needs to be revised.  By the “work environment”, perhaps it is meant “in the work environment”, and the word “forced”, first of all, has no relevance to the concept of deception. It may be better to “encourage you” instead of “forcing”  Insistence of officials to have an attractive and seductive appearance and clothing in the workplace to attract customers. | 0.28 | - | It was removed due to low CVR. | - |
| 38. Forced to establish an unusual relationship to maintain working conditions. | It is not clear, if you mean Forced by colleagues, mention it. | 1 | 1 | It remained unchanged with the opinion of the research team. | 4.91 |
| 39. Threats of demotion or dismissal for refusing to have sex. | It is not clear if you mean coercion by colleagues  In all options, replace the word sexual cooperation with a more expressive and clear word.  The word threat at the beginning of this item seems to be inconsistent with the concept of deception, and since item 40 covers this item in reverse, it should be deleted or revised. | 0.85 | - | It was removed with the opinion of the research team. | - |
| 40. Offer a job promotion in exchange for sexual willingness | It is not clear. If you mean forced by colleagues, mention it.  Items 40 and 42 can be merged | 1 | - | It was removed with the opinion of the research team. | - |
| 41. Misbehavior due to refusal of sexual willingness. | Misbehavior seems to be different from the concept of seduction. It is better to revise or delete the item. | 0.85 | - | It was removed with the opinion of the research team. | - |
| 42. A tempting financial or professional offer in exchange for sexual willingness | It overlaps with item 35 and 40, it is better to delete it. | 0.57 | 0.92 | It remained unchanged. | 5 |
| **Cyber sexual harassment** |  |  |  |  |  |
| 43. Sending you text messages with sexual content | - | 1 | 1 | It remained unchanged. | 5 |
| 44. Sending vulgar and sexy photos and videos to you through social networks. | 44 and 46 can be merged.  Sending you vulgar photos and videos and links through social networks | 1 | 1 | Sending or showing you vulgar photos and videos and links through social networks and email | 5 |
| 45. Threats to publish photos, videos, and private chats if there is no sexual willingness | - | 0.85 | 1 | It remained unchanged. | 5 |
| 46. Sending pornographic links to you via email or social media | Can be merged with item 44. | 0.71 | - | It was removed with the opinion of the research team. | - |
| 47. Asking you to send a nude photo of a part your body | It is not in this area, it is verbal. | 0.85 | 1 | It remained unchanged with the opinion of the research team. | 5 |
| 48. Sending you a nude photo of their body | - | 0.85 | 1 | It remained unchanged. | 5 |
